# Ligand-bound Structures and Site-directed Mutagenesis Identify the Acceptor and Secondary Binding Sites of *Streptomyces coelicolor* Maltosyltransferase GlgE*

**DOI:** 10.1074/jbc.M116.748160

**Published:** 2016-08-16

**Authors:** Karl Syson, Clare E. M. Stevenson, Farzana Miah, J. Elaine Barclay, Minhong Tang, Andrii Gorelik, Abdul M. Rashid, David M. Lawson, Stephen Bornemann

**Affiliations:** From the Biological Chemistry Department, John Innes Centre, Norwich Research Park, Norwich NR4 7UH, United Kingdom

**Keywords:** carbohydrate-binding protein, crystal structure, glycosyltransferase, oligosaccharide, site-directed mutagenesis

## Abstract

GlgE is a maltosyltransferase involved in α-glucan biosynthesis in bacteria that has been genetically validated as a target for tuberculosis therapies. Crystals of the *Mycobacterium tuberculosis* enzyme diffract at low resolution so most structural studies have been with the very similar *Streptomyces coelicolor* GlgE isoform 1. Although the donor binding site for α-maltose 1-phosphate had been previously structurally defined, the acceptor site had not. Using mutagenesis, kinetics, and protein crystallography of the *S. coelicolor* enzyme, we have now identified the +1 to +6 subsites of the acceptor/product, which overlap with the known cyclodextrin binding site. The sugar residues in the acceptor subsites +1 to +5 are oriented such that they disfavor the binding of malto-oligosaccharides that bear branches at their 6-positions, consistent with the known acceptor chain specificity of GlgE. A secondary binding site remote from the catalytic center was identified that is distinct from one reported for the *M. tuberculosis* enzyme. This new site is capable of binding a branched α-glucan and is most likely involved in guiding acceptors toward the donor site because its disruption kinetically compromises the ability of GlgE to extend polymeric substrates. However, disruption of this site, which is conserved in the *Streptomyces venezuelae* GlgE enzyme, did not affect the growth of *S. venezuelae* or the structure of the polymeric product. The acceptor subsites +1 to +4 in the *S. coelicolor* enzyme are well conserved in the *M. tuberculosis* enzyme so their identification could help inform the design of inhibitors with therapeutic potential.

## Introduction

**FIGURE 1.**
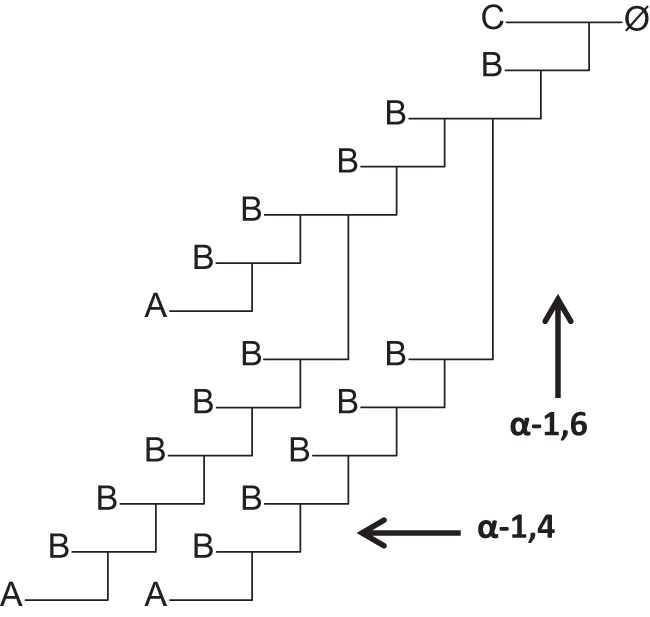
**Constituent chains of α-glucan.** The C chain retains the only reducing end glucose residue (∅) within a polymer molecule. B chains bear branches, whereas A chains do not.

GlgE is an α-maltose 1-phosphate:(1→4)-α-d-glucan 4-α-d-maltosyltransferase (starch synthase (maltosyl-transferring), EC 2.4.99.16) (1, 2) and a member of the carbohydrate-active enzymes database (CAZy) GH13_3 family (3). It is the third and defining enzyme of the four-step GlgE pathway that is responsible for the biosynthesis of a branched α-glucan (4). This pathway involves the sequential conversion of trehalose into α-maltose (5); α-maltose 1-phosphate (6), an α-1,4-linked α-glucan (1, 2); and finally an α-1,4-linked α-glucan with α-1,6-linked branches (7). The polymer comprises large dendrimers tens of nm in diameter (7). Each polymer molecule possesses a single C chain that bears at least one branch and the sole reducing end (Fig. 1). B chains are branches that themselves bear branches, whereas A chains do not such that A chains are largely at the periphery of the polymer. GlgE-derived α-glucan resembles glycogen except that it comprises shorter linear chains, has a correspondingly higher proportion of α-1,6 linkages, and has relatively fewer A chains. It is the combination of GlgE and the branching enzyme GlgB that defines the structure of the polymer. The key contribution that GlgE makes is to preferentially extend A chains, limiting the opportunity for GlgB to introduce a second branch into B and C chains. However, the structural basis for such specificity was not understood.

The GlgE pathway is widespread among bacteria (8) and has been best characterized in actinomycetes such as streptomycetes (9) and mycobacteria (10). *Mycobacterium tuberculosis* GlgE has attracted particular attention because of the demonstration that it is a genetically validated drug target (11). Unusually, the killing mechanism involves the hyperaccumulation of α-maltose 1-phosphate to toxic levels rather than the blocking of the production of α-glucan. Toxicity results in pleiotropic effects, and the target of this metabolite remains elusive. Driven by the desire to develop new therapies to tackle tuberculosis (11), some inhibitors of GlgE have been described. For example, substrate and transition state analogues have been reported with IC_50_/*K_i_* values of >200 μm (12, 13). Other potential inhibitors have been proposed based on theoretical docking calculations (14–16).

**FIGURE 2.**
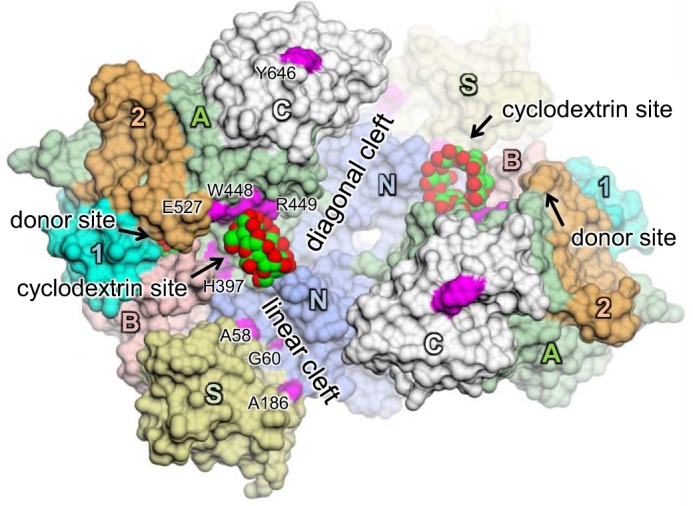
**Structural overview of *S. coelicolor* GlgE isoform 1.** A molecular surface representation is shown of a previously solved structure with maltose and α-cyclodextrin bound (Protein Data Bank accession code 3ZT6) (19). The subunits of the dimer interact head to tail and are each composed of domains A (*green*), B (*pink*), C (*gray*), N (*blue*), and S (*gold*) and inserts 1 (*cyan*) and 2 (*orange*) as indicated. Maltose occupies the donor site of domain A, whereas α-cyclodextrin binds to a hydrophobic ridge comprising domain A of one subunit and domain N of the other. Linear and diagonal clefts radiate away from the cyclodextrin site. Amino acids mutated in this study are highlighted in *magenta*. Oxygen atoms are shown in *red*, and carbon atoms are shown in *green* for the carbohydrate ligands.

A number of crystal structures of GlgE have been solved, which will assist in the development of inhibitors. A high resolution structure of the *M. tuberculosis* enzyme has proven difficult to obtain (17), so most work has been done with *Streptomyces coelicolor* GlgE isoform 1 (18–20), which has very similar properties. Additional structures of the *Mycobacterium thermoresistibile* enzyme have also been reported recently (21), providing an alternative model for the *M. tuberculosis* enzyme. The enzyme consists of five domains (Fig. 2), four of which are typical of the α-amylase family (22). The catalytic domain A is elaborated by domain B, which forms a lid over the donor site, and inserts 1 and 2. Domain N forms the central core of the protein and much of the interface between the two subunits of the biological dimer. Domain C of the *M. tuberculosis* enzyme is capable of binding a malto-oligosaccharide close to the interface with domain A but remote from the active site (17). There are other examples of α-amylase enzymes with a secondary binding site on domain C (23), so this appears to be an evolutionarily conserved feature. Finally, domain S, which is unusual in this family, is able to adopt two conformations that seem to influence the ability of the domain B lid on the neighboring subunit to open and/or close (21). The GlgE dimer forms two distinct clefts on the same face of the structure as the active sites. In addition, there are several surface Ser and Thr residues in the B, S, and N domains of the *M. tuberculosis* enzyme that are subject to phosphorylation by the kinase PknB, leading to negative regulation (24). These residues are distinct from the known glucan binding sites. Therefore, regulation may be mediated through long range electrostatic effects on catalysis and/or on the ability of the donor site lid to open and close.

The donor site has been structurally characterized using wild-type and mutated forms of GlgE with either maltose (17, 19, 21), α-maltose 1-phosphate (18), or substrate/transition state analogues bound (17, 20). Not only do we have a clear picture of how the donor binds to the enzyme but also the nature of a covalently linked glycosyl-enzyme intermediate produced in the next step in the catalytic cycle (18). Indeed, it has been unequivocally demonstrated that Asp-394 (*S. coelicolor* numbering) is the nucleophile that attacks the donor to liberate inorganic phosphate by trapping a β-maltosyl-enzyme intermediate. A neighboring Glu-423 acid/base catalytic side chain is well placed to assist by protonating the phosphate leaving group and then deprotonating an incoming acceptor substrate. Loss of activity resulting from mutation of Glu-423 supports this role in catalysis (18). We therefore have a good understanding of the structures of the first two intermediates of what is a retaining double displacement mechanism consistent with other members of the GH13 family of enzymes. Conversely, the acceptor site has not been clearly defined in any studies to date. It is known that a hydrophobic ridge between two clefts is capable of binding cyclodextrins and that the *S. coelicolor* enzyme is inhibited by cyclodextrins by competing with acceptors (19). Therefore, the acceptor site probably overlaps with the cyclodextrin site. However, this site is over 10 Å away from the donor site and oriented orthogonally with respect to the donor, making modeling of the entire acceptor site challenging.

Important issues that remain unresolved therefore include how an acceptor binds such that it prefers A chains (Fig. 1) and what role the secondary binding site has. To address these questions, we have used fresh approaches with mutated forms of *S. coelicolor* GlgE isoform 1 to assess the role of specific amino acid side chains in enzyme activity and to obtain new structures with oligosaccharides bound.

## Results

### 

#### 

##### Mapping the Acceptor Binding Site

There are two conspicuous clefts that are adjacent to the donor binding site (Fig. 2). The one closest to the donor site is the linear cleft that is formed primarily by domains S and N of the neighboring subunit. To establish whether linear acceptor substrates of GlgE can bind along this linear cleft, we mutated three solvent-exposed amino acid side chains (Ala-58, Gly-60, and Ala-186) along its length to Arg. The substitution with such a large side chain would be expected to compromise the binding of acceptors if the linear cleft were to overlap with the acceptor binding site. By monitoring the production of inorganic phosphate with malachite green, the kinetics of each of the three variants was tested using maltohexaose as the acceptor. In each case, little difference in the kinetics was observed compared with the wild-type enzyme (Table 1). It would therefore seem that short linear acceptors do not bind in the linear cleft. Similarly, when these GlgE variants were assayed with α-glucan polymer isolated from *Streptomyces venezuelae* rather than maltohexaose, their values of *k*_cat_^app^/*K*_*m*_^app^ were less than 2-fold different from that of the wild-type enzyme (data not shown), suggesting that the linear cleft is not particularly important in the accommodation of the larger acceptor either.

**TABLE 1:** **Kinetics of GlgE variants with maltohexaose as the donor and 0.25 mm α-maltose 1-phosphate**

GlgE	*k*_cat_^app^	*K*_*m*_^app^	*k*_cat_^app^/*K*_*m*_^app^
	*s*^−*1*^	*mm*	*m*^−*1*^ *s*^−*1*^
**Wild type**	9.4 ± 0.5	0.49 ± 0.07	19000 ± 3000

**Linear cleft**			
A58R	7.4 ± 0.5	0.50 ± 0.09	14800 ± 2800
G60R	7.2 ± 0.4	0.49 ± 0.07	15000 ± 2200
A186R	9.2 ± 0.5	0.60 ± 0.08	15300 ± 2200

**Acceptor site**			
W448A	ND*^a^*	ND	94 ± 10
W448E	ND	ND	75 ± 6
R449A	9.6 ± 0.8	6.7 ± 1.0	9600 ± 1600
H397R	0.220 ± 0.002	0.36 ± 0.01	580 ± 14
E527A	7.2 ± 0.5	0.32 ± 0.07	22500 ± 2100
E527R	6.5 ± 0.3	0.52 ± 0.07	12500 ± 1800

**Secondary site**			
Y646A	9.3 ± 0.6	0.66 ± 0.11	14000 ± 2500

*^a^* ND, not derived due to lack of enzyme activity.

We have previously shown that cyclodextrins bind to a largely hydrophobic ridge between the linear and diagonal clefts over 10 Å from the donor site (Fig. 2) (19). Because cyclodextrins inhibit *S. coelicolor* GlgE competitively with regard to acceptor substrates, the acceptor binding site might be expected to overlap with this site. Significant interactions between cyclodextrins and GlgE involve the side chains of Trp-448 and Arg-449, so we explored the effect of mutations at these positions. When Trp-448 was substituted by either Ala or Glu, the enzyme lost almost all of its detectable activity (Table 1), consistent with the acceptor binding at this position through van der Waals interactions. When Arg-449 was mutated to Ala, the *K*_*m*_^app^ for maltohexaose increased 14-fold with no change in the value of *k*_cat_^app^, showing that binding was somewhat compromised by the loss of some hydrogen bonding interactions. These observations provide additional evidence that the acceptor binding site overlaps with the cyclodextrin binding site.

The considerable distance between the cyclodextrin and donor binding sites necessitates several sugar residues to bridge the gap (Fig. 2). To obtain some indication which path the acceptor binding site takes, we mutated two amino acid side chains that flank either side of the direct path between the two sites. We first substituted His-397 to Arg and found that *k*_cat_^app^ was reduced 43-fold (Table 1), strongly suggesting that the orientation of the acceptor is compromised in its ability to attack the glycosyl-enzyme intermediate of this form of the enzyme. The associated *k*_cat_^app^/*K*_*m*_^app^ was consequently reduced 33-fold. By contrast, mutation of Glu-527 to Ala had relatively little effect, suggesting that the Glu side chain at this position does not have a significant role in either binding or orienting the acceptor. However, introduction of an Arg at this position did lower the *k*_cat_^app^/*K*_*m*_^app^ for the acceptor by about a third, suggesting that the bulkier side chain had a greater reach, leading to a slight distortion of the orientation of the acceptor. Nevertheless, the effect of this substitution was much smaller than the equivalent mutation of His-397.

The acceptor chain length specificity of each of the mutants was evaluated. In each case, the optimum was between five and seven glucose residues in length. This compares with six for the wild-type protein. The variants therefore showed only minor changes in chain length specificity with no obvious trends emerging. Nevertheless, it is clear from the kinetic constants with maltohexaose that the acceptor binding site overlaps with the cyclodextrin binding site and passes by His-397.

##### The Cyclodextrin Site Can Bind a Linear Malto-oligosaccharide

**FIGURE 3.**
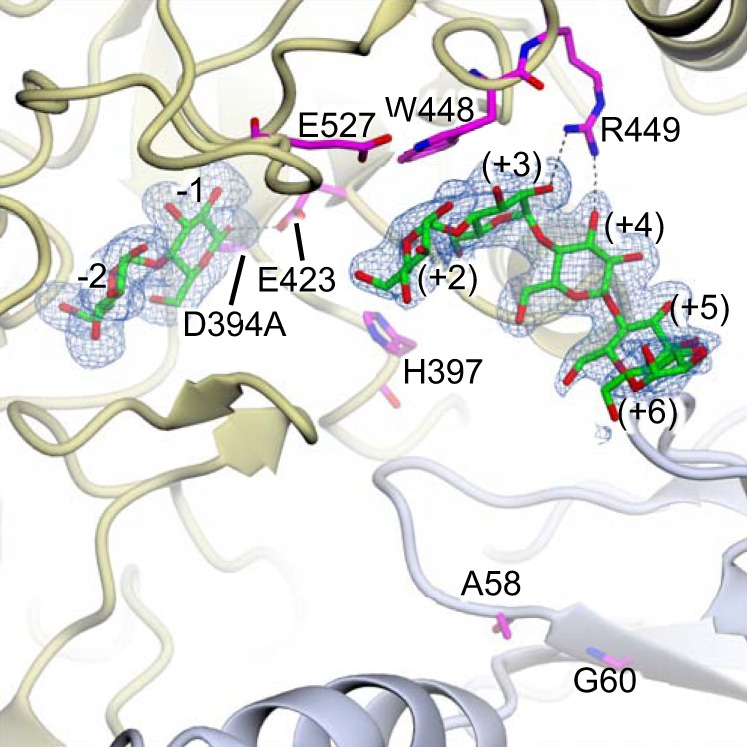
**A linear malto-oligosaccharide can bind to the cyclodextrin binding site.** Co-crystallization of a D394A variant of GlgE with maltodextrin yielded a structure with maltose bound to the donor site and maltopentaose bound to the cyclodextrin binding site as indicated by the omit electron density map (1.95-Å resolution; contoured at ∼3.0 σ). The residues of the maltopentaose superpose well with those of cyclodextrins in complex with GlgE (Protein Data Bank accession codes 3ZST, 3ZT6, and 3ZT7) (19). There is less correspondence with the acceptor subsites seen in the product-bound structures described here (Fig. 4); for this reason, the subsite labels are shown in *parentheses* (see also Fig. 9). Interactions between the maltopentaose and GlgE involved (from *left* to *right*) Trp-448, Arg-449, Tyr-445, Pro-428, Arg-427, and Thr-426 (backbone CO) from one subunit and Gly-84 and Pro-83 from the neighboring subunit. The two subunits are distinguished by *gold* and *slate* coloring, oxygen atoms are shown in *red*, carbon atoms are shown in *green* for the malto-oligosaccharide, and the amino acids mutated in this study are shown in *magenta*. The orientation of GlgE is identical to that of Fig. 2.

Attempts to identify the acceptor binding site using protein crystallography have previously been thwarted by the ability of GlgE to disproportionate and ultimately hydrolyze malto-oligosaccharides to form maltose. To prevent GlgE from catalyzing such reactions, a D394A GlgE variant that lacks the nucleophilic side chain (18) was generated. This GlgE variant was co-crystallized (Table 2) with a preparation of maltodextrin, which contains a wide range of both linear and α-1,6 branched oligosaccharides according to mass spectrometry and NMR spectroscopy. The donor binding site was occupied by maltose as has been observed in many of the published structures. However, a linear malto-oligosaccharide was observed bound to the cyclodextrin binding site for the first time (Fig. 3). Five sugar residues were resolved, four of which occupied very similar positions to those observed with β-cyclodextrin such that they interacted with Trp-448 and Arg-449. The remaining glucose residue was at the non-reducing end and was slightly displaced such that it was more oriented toward the donor binding site. However, this glucose residue was still over 10 Å away from the donor site. Therefore, the complete acceptor site was not defined in this structure.

**TABLE 2:** **Summary of x-ray data and model parameters for *S. coelicolor* GlgE isoform 1 complexes** Values in parentheses are for the outer resolution shell. r.m.s., root mean square; —, no other ligands.

Data set	E423A + maltoheptaose	E423A + malto-octaose	D394A + maltodextrin
**Data collection**			
Beamline	I04-1	I04-1	I24
Wavelength (Å)	0.920	0.920	0.978
Detector	Pilatus 2 m	Pilatus 2 m	Pilatus 6 m
Resolution range (Å)	62.83–2.30 (2.36–2.30)	49.92–2.50 (2.56–2.50)	81.40–1.95 (2.00–1.95)
Space group	P4_1_2_1_2	P4_1_2_1_2	P4_1_2_1_2
Cell parameters (Å)	*a* = *b* = 114.08, *c* = 314.14	*a* = *b* = 113.61, *c* = 313.69	*a* = *b* = 115.11, *c* = 311.36
Total no. of measured intensities	1,708,887 (119,311)	938,920 (42,909)	1,934,155 (27,203)
Unique reflections	92,902 (6,680)	72,101 (5,252)	147,828 (8,730)
Multiplicity	18.4 (17.9)	13.0 (8.2)	13.1 (3.1)
Mean *I*/σ(*I*)	27.6 (3.5)	17.9 (2.3)	17.5 (1.2)
Completeness (%)	99.9 (99.4)	100.0 (100.0)	97.1 (79.9)
*R*_merge_*^a^*	0.068 (0.998)	0.091 (0.846)	0.083 (0.837)
*R*_meas_*^b^*	0.071 (1.056)	0.098 (0.967)	0.086 (1.081)
CC_1/2_*^c^*	0.999 (0.868)	0.999 (0.628)	0.999 (0.589)
Wilson *B* value (Å^2^)	47.0	50.9	26.1

**Refinement**			
Resolution range (Å)	62.83–2.30 (2.36–2.30)	49.92–2.50 (2.56–2.50)	81.40–1.95 (2.00–1.95)
Reflections: working/free*^d^*	88,145/4,636	68,441/3,560	140,419/7,409
*R*_work_/*R*_free_*^e^*	0.181/0.209 (0.252/0.272)	0.179/0.209 (0.314/0.361)	0.177/0.194 (0.349/0.365)
Ramachandran plot: favored/allowed/disallowed*^f^* (%)	98.5/1.3/0.2	97.8/2.1/0.1	98.1/1.6/0.3
r.m.s. bond distance deviation (Å)	0.011	0.010	0.010
r.m.s. bond angle deviation (°)	1.44	1.43	1.40
No. of protein residues: chain A/chain B (ranges)	649 (15–663)/649 (15–663)	649 (15–663)/649 (15–663)	648 (15–662)/648 (15–662)
No. of sugars/water molecules/other ligands	24/484/0	26/447/0	29/1,092/5
Mean *B* factors: protein/sugars/water/other/overall (Å^2^)	61/80/53/—/61	67/93/54/—/67	34/56/37/62/35

**Protein Data Bank accession code**	5CVS	5LGV	5LGW

*^a^ R*_merge_ = Σ*_hkl_*Σ*_i_*|*I_i_*(*hkl*) − 〈*I*(*hkl*)〉|/Σ*_hkl_*Σ*_i_I_i_*(*hkl*).

*^b^ R*_meas_ = Σ*_hkl_*[*N*/(*N* − 1)]^1/2^ × Σ*_i_*|*I_i_*(*hkl*) − 〈*I(hkl*)〉|/Σ*_hkl_*Σ*_i_I_i_*(*hkl*) where *I_i_*(*hkl*) is the *i*th observation of reflection *hkl*, 〈*I*(*hkl*)〉 is the weighted average intensity for all observations *i* of reflection *hkl*, and *N* is the number of observations of reflection *hkl*.

*^c^* CC_1/2_ is the correlation coefficient between symmetry-equivalent intensities from random halves of the data set.

*^d^* The data set was split into “working” and “free” sets consisting of 95 and 5% of the data, respectively. The free set was not used for refinement.

*^e^* The *R* factors *R*_work_ and *R*_free_ were calculated as follows: *R* = Σ(|*F*_obs_ − *F*_calc_|)/Σ|*F*_obs_| where *F*_obs_ and *F*_calc_ are the observed and calculated structure factor amplitudes, respectively.

*^f^* As calculated using MolProbity (43).

##### A Product-bound Structure

**FIGURE 4.**
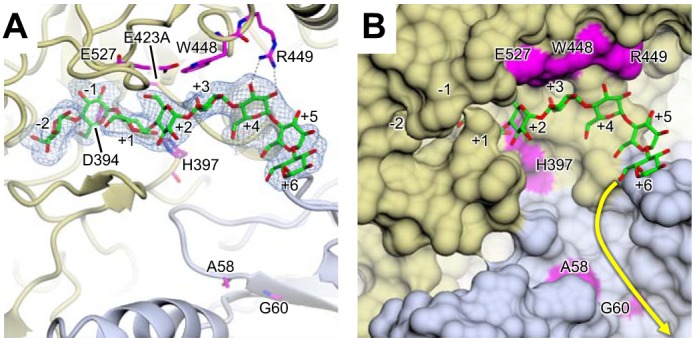
**A product-bound structure.** Soaking a crystal of the E423A variant of GlgE with malto-octaose and 2-deoxy-2-fluoro-α-maltosyl fluoride resulted in only the former being detected in the structure. Malto-octaose was bound to both the donor and cyclodextrin binding sites. Each subsite from −2 to +6 is highlighted along with the amino acids mutated in this study. *A*, malto-octaose was clearly resolved as indicated by the omit electron density map (2.5-Å resolution; contoured at ∼3.0 σ). It was bound to the subsites −2 and −1 of the donor site (18, 19) and +1 to +6 of what would be the acceptor site. The locations of the catalytic nucleophile (Asp-394) and acid/base amino acid (Glu-423 mutated to Ala in this case) are indicated. *B*, when the protein is depicted with a molecular surface, it is apparent that the C6 primary hydroxyl groups of the glucose residues project toward the protein surface in subsites +1 to +5. By contrast, it is feasible that an α-1,6-linked malto-oligosaccharide could emanate from the +6 subsite and be accommodated by the linear cleft as indicated by the *yellow arrow*. In each panel, the two subunits are distinguished by *gold* and *slate* coloring, oxygen atoms are shown in *red*, and carbon atoms are shown in *green* for the malto-oligosaccharide and in *magenta* for amino acids mutated in this study. The orientation of GlgE is identical to that of Fig. 2.

Co-crystallization and soaks of a different catalytically compromised E423A variant of GlgE, which lacks the acid/base catalytic side chain (18), were carried out with various substrates and/or substrate analogues. In an attempt to obtain a structure of a trapped maltosyl-enzyme intermediate (18) with an acceptor bound in a productive conformation, we soaked a crystal of the E423A variant of GlgE in both malto-octaose and 2-deoxy-2-fluoro-α-maltosyl fluoride. Fortuitously, this yielded a structure where malto-octaose was bound such that it occupied both the donor and cyclodextrin binding sites (Fig. 4), whereas the donor analogue was unexpectedly absent from the crystal. This structure would therefore appear to represent a product-bound species with subsites −2 to +6 occupied. The two sugar residues in the donor site are oriented in a manner similar to those observed with maltose and α-maltose 1-phosphate (18, 19). The residues occupying subsites +3 to +6 overlap with those seen in cyclodextrins and the maltopentaose described above but are somewhat displaced to allow the product to bind in the appropriate orientation. The maltopentaose interacts with Trp-448 (stacking to the +3 sugar), Arg-449 (3.1-Å H-bonds to O2 and O3 of the +4 sugar), and His-397 (3.2-Å H-bond to O2 of the +1 sugar) but not Glu-527 (>4.7-Å O-O distance) as expected from the kinetics of the variants described above. The conformation of the oligosaccharide was unusually flat and extended (Table 3) compared with amylose or amylopectin (25), which have α-1,4 torsion angles closer to that of maltose (ϕ = 116° and ψ = −118°) (26). This was particularly striking at the linkage where the donor and acceptor glucose residues occupying subsites −1 and +1 connect (ϕ = 34° and ψ = −157°), giving the longest distance between the 2- and 3′-hydroxyl oxygens of 4.9 Å (Table 3). This likely reflects the geometry required to allow glycosyl transfer to occur. An essentially identical structure was also obtained by soaking a crystal with maltoheptaose except that the +6 subsite was not occupied. This showed that it was possible to obtain a product-bound structure in the absence of a donor substrate analogue.

**TABLE 3:** **Conformation of α-1,4 linkages in saccharides bound to *S. coelicolor* GlgE isoform 1**

Glucose residue	Subsite	2-3′*^a^*	O5-C1-O1-C4′*^b^* ϕ	C1-O1-C4′-C5′*^b^* ψ
		Å	°	°
**Crystalline α-maltose*^c^***	NA*^d^*	2.8	116	−118

**Malto-octaose (product)**				
1, non-reducing end	−2	4.0	77	−151
2	−1	4.9	34	−157
3	+1	4.7	48	−156
4	+2	4.2	59	−152
5	+3	4.1	68	−140
6	+4	3.2	100	−117
7	+5	3.1	100	−121
8, reducing end	+6	NA	NA	NA

*^a^* The distances between the oxygen atoms at the 2- and 3′-positions of adjacent non-reducing and reducing end residues, respectively, are shown.

**FIGURE 5.**
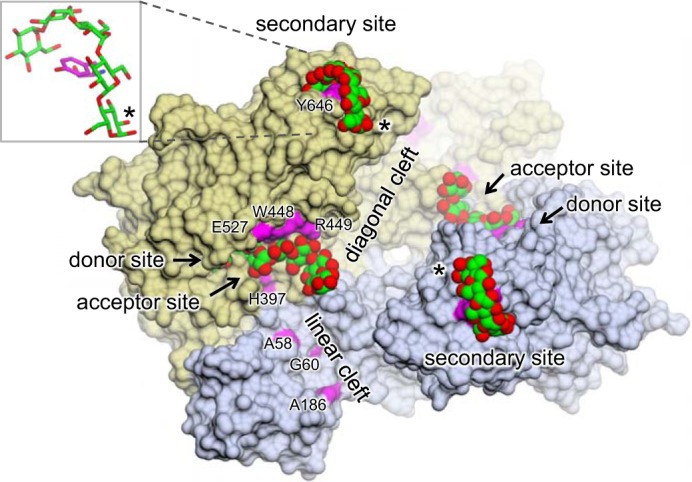
***S. coelicolor* GlgE has an unexpected secondary binding site.** The product-bound structures of the E423A variant of GlgE had a malto-oligosaccharide bound to a secondary site in each subunit. Only five glucose residues were resolved despite the structures being obtained with either malto-octaose (as shown here) or maltoheptaose. The glucose residue at the reducing end (highlighted with an *asterisk*) is flipped relative to the other residues. The two subunits are distinguished by *gold* and *slate* coloring, oxygen atoms are shown in *red*, and carbon atoms are shown in *green* for the malto-oligosaccharide and in *magenta* for amino acids mutated in this study. The orientation of GlgE is identical to that of Fig. 2.

*^b^* The torsion angles (as defined by Damager *et al.* (26)) relate to the structure shown in Figs. 4 and 5.

*^c^* The values associated with crystalline α-maltose are shown for comparison (44).

*^d^* NA, not applicable.

##### A Newly Identified Secondary Binding Site in S. coelicolor GlgE

The product-bound structures of the E423A variant of GlgE had an additional malto-oligosaccharide bound at a secondary site located on domain C (Fig. 5). Unexpectedly, this is a previously undescribed secondary binding site over 40 Å away from the donor site and over 10 Å away from that recently described for the *M. tuberculosis* enzyme (17). In each case, five glucose residues were well resolved, clearly showing that they that were wrapped around Tyr-646 of *S. coelicolor* GlgE. The non-reducing end of this malto-oligosaccharide occupied a pocket on the surface of the enzyme, precluding the ability to accommodate any more glucose residues at this location. By contrast, the reducing end glucose residue was flipped relative to the other residues such that its α-anomeric hydroxyl projected into solvent (Fig. 5). The potential for the malto-oligosaccharide to extend into solvent was supported by the detection of additional electron density suggesting the existence of at least one other glucose residue. However, it was not possible to resolve additional residues unambiguously. The likelihood is that malto-octaose/maltoheptaose was bound to this site but that three/two glucose residues at the reducing end were not well ordered in the crystal. If so, this implies that the secondary binding site is capable of accommodating linear malto-oligosaccharides with a length of greater than five residues.

**FIGURE 6.**
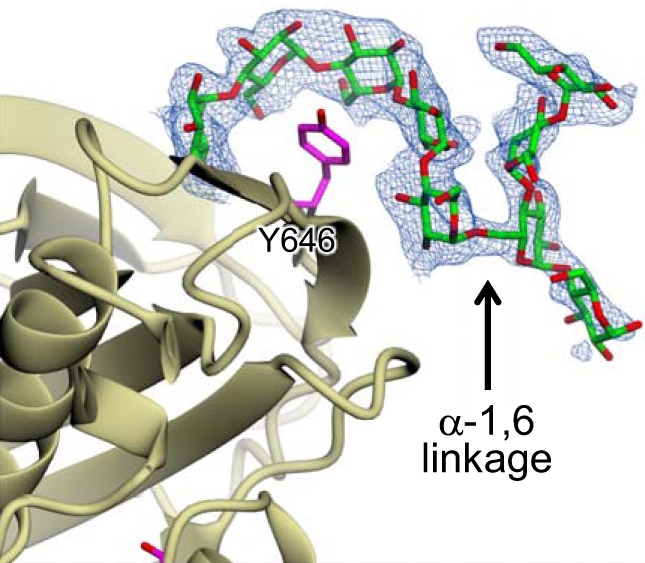
**The secondary binding site of *S. coelicolor* GlgE can bind a branched oligosaccharide.** A branched malto-oligosaccharide comprising a proximal pentasaccharide A chain and a distal tetrasaccharide C chain were bound to the secondary binding site as shown by the omit electron density map (1.95-Å resolution; contoured at ∼3.0 σ). The C chain made no contact within the biologically relevant GlgE dimer. Oxygen atoms are shown in *red*, and carbon atoms are shown in *green* for the malto-oligosaccharide and in *magenta* for Tyr-646. The orientation of GlgE has been altered slightly relative to Fig. 2 for clarity.

Similarly, another oligosaccharide was observed in the structure of the D394A GlgE variant obtained with maltodextrin. Again, five glucose residues of an α-1,4-linked malto-oligosaccharide were wrapped around the side chain of Tyr-646 with the reducing end glucose residue being flipped relative to the others (Fig. 6). Remarkably, several additional glucose residues were resolvable within the electron density. In one copy of the GlgE subunit within the crystal, an additional four sugar residues were present that made no direct contact with this subunit but instead interacted with a subunit from a neighboring GlgE homodimer in the crystal lattice. There was an α-1,6 linkage between the reducing end of the proximal pentasaccharide and the second residue from the reducing end of the distal tetra-saccharide. Therefore, the protein-bound pentasaccharide constitutes an A chain emanating from a tetrasaccharide C chain. In the other copy of the GlgE subunit, only the first additional α-1,6-linked distal sugar residue was resolvable.

##### The Secondary Binding Site Kinetically Assists the Extension of Polymers but Is Not Essential

To establish the role of the secondary binding site of *S. coelicolor* GlgE, we generated a Y646A variant that would disrupt the binding of oligosaccharides to this site. The kinetic constants of this variant were very similar to those of the wild-type enzyme when using maltohexaose as the acceptor substrate (Table 1). Perhaps this is to be expected because the secondary binding site is quite remote from the donor and acceptor sites, and initial rates are measured in the enzyme assay when oligosaccharides have not yet reached significant lengths.

To test whether the secondary binding site becomes important when GlgE extends a polymer, the assays were repeated using α-glucan. In this case, it was clear that disruption of the secondary binding site had an impact on activity. The value *k*_cat_^app^ went down, compared with the wild-type enzyme, from 3.5 ± 0.2 to 0.85 ± 0.07 s^−1^, whereas that of *K*_*m*_^app^ went up from 0.33 ± 0.05 to 2.5 ± 0.4 mg ml^−1^. Thus, the value of *k*_cat_^app^/*K*_*m*_^app^ decreased 31-fold.

**FIGURE 7.**
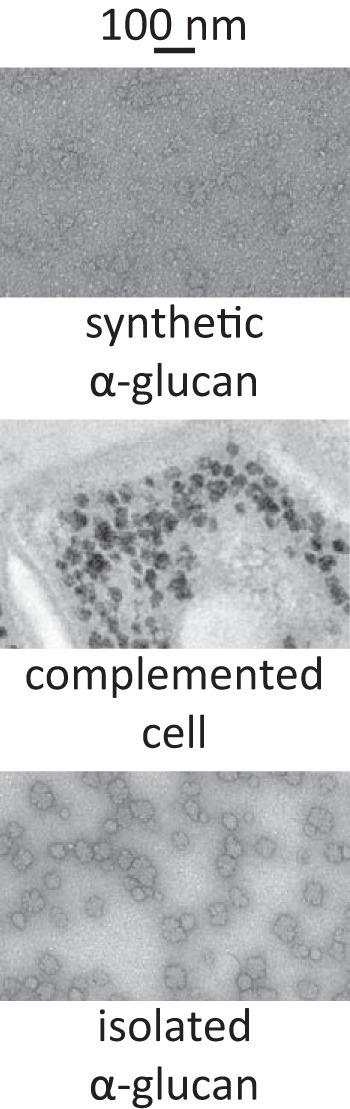
**Disruption of the secondary binding site of *Streptomyces* GlgE has no effect on α-glucan particles generated *in vitro* and *in vivo*.** Synthetic α-glucan particles were generated using the *S. coelicolor* GlgE Y646A variant, *S. coelicolor* branching enzyme GlgB isoform 1, α-maltose 1-phosphate, and maltohexaose and imaged using electron microscopy with a negative uranyl acetate stain. An *S. venezuelae glgE*-null mutant was complemented with an *S. venezuelae glgE* gene coding for a GlgE Y665A variant (equivalent to *S. coelicolor* Y646A) and imaged with a positive periodic acid-thiocarbohydrazide-silver proteinate stain. α-Glucan isolated from the complemented strain was imaged with the negative stain.

The impact of the secondary binding site on the linear chain lengths of a branched polymer was then assessed. The *S. coelicolor* GlgE Y646A variant was mixed with the *S. coelicolor* branching enzyme GlgB isoform 1 and exposed to α-maltose 1-phosphate and maltohexaose to generate α-glucan polymer. The resulting particles resembled those obtained with the wild-type enzyme (Fig. 7) (7). Debranching of the polymer with isoamylase allowed the constituent linear chain length to be determined using mass spectrometry. It was apparent that the GlgE variant, in combination with GlgB, generated linear chain lengths (mean and chain length distributions of 8.3 ± 2.8) indistinguishable from those of the wild-type enzyme (7.9 ± 2. 8) in back-to-back experiments. Therefore, the secondary binding site does not seem to have a role in defining the structure of the polymer *in vitro*.

**FIGURE 8.**
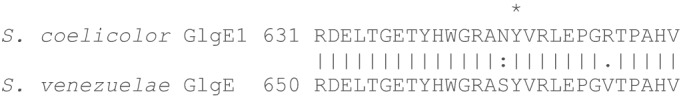
**Conservation of the secondary binding site between *S. coelicolor* and *S. venezuelae*.** A partial amino acid sequence alignment is shown between *S. coelicolor* GlgE isoform 1 and *S. venezuelae* GlgE. The number of the first amino acid of each partial sequence is indicated. Tyr-646 and Tyr-665 of the secondary binding sites of *S. coelicolor* and *S. venezuelae* enzymes, respectively, are highlighted with the *asterisk*. The *vertical lines* indicate amino acid identity, whereas the *colon* and *period* indicate decreasing degrees of amino acid similarity.

*S. coelicolor* possesses two copies of the *glgE* gene, making the genetic manipulation of the GlgE pathway in this organism less tractable than in the closely related *S. venezuelae*, which has only one copy (8, 9). The GlgE homologues from *S. coelicolor* (isoform 1) and *S. venezuelae* share 79% amino acid sequence identity together with very high conservation around the secondary binding site including the key Tyr residue (Fig. 8). It would therefore be expected that the *S. venezuelae* enzyme also possesses the secondary binding site and that the Tyr to Ala mutation would disrupt the function of this site. To determine whether the secondary binding site is required for the function of GlgE *in vivo*, a *glgE*-null mutant of *S. venezuelae* (9) was complemented with an *S. venezuelae glgE* gene coding for the equivalent Tyr to Ala GlgE variant (Y665A). Without complementation with an intact *glgE* gene, the mutant strain is known not to produce α-glucan and to exhibit a delayed growth phenotype due to the accumulation of α-maltose 1-phosphate. However, the strain complemented with the GlgE variant grew normally and produced α-glucan in a manner similar to the wild-type strain (Fig. 7). This suggests that the secondary binding site is not essential *in vivo* in the conditions tested despite a potential loss of activity.

α-Glucan isolated from the complemented strain resembled that isolated from the wild type according to electron microscopy (Fig. 7). The mean chain length of the polymer from the complemented mutant strain (7.9 ± 2.0) was similar to that isolated from the wild type (7). In addition, the degree of digestion by β-amylase (19%) was also comparable with that of the wild-type polymer (21.0 ± 4.7%). Therefore, the secondary binding site does not seem to be important in defining the gross or fine structure of the polymer.

## Discussion

### 

#### 

##### Acceptor Binding Site

**FIGURE 9.**
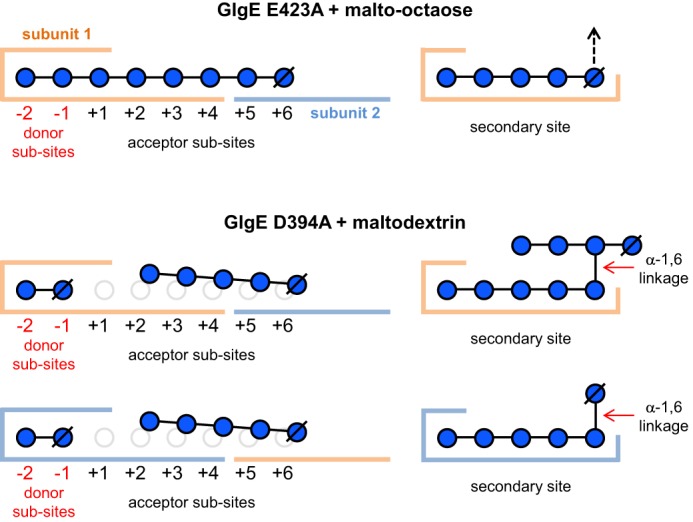
**Schematic summarizing the carbohydrate binding sites of *S. coelicolor* GlgE identified in this study.** The GlgE variants and the oligosaccharides bound to them are shown where each protein subunit is colored in *gold* or *slate*, glucose residues are shown as *blue circles*, carbohydrate linkages are all α-1,4 unless indicated otherwise, and reducing end glucose residues are indicated by the ∅ symbol. The same ligands were visible in each of the two subunits of the GlgE E423A variant, whereas the occupancies of the secondary sites differed in the D394A variant. The *dashed arrow* indicates the likelihood that an α-1,4-linked malto-oligosaccharide extends further into solvent.

The key aim of this study was to identify the acceptor binding site. By using mutagenesis to prevent GlgE from slowly hydrolyzing malto-oligosaccharides, it was possible to obtain a structure with malto-octaose bound to the active site (Figs. 4, 5, and 9). The occupancy of the donor subsites −2 and −1 together with subsites +1 to +6 indicated that this represents a product-bound structure that reveals the locations of the first six acceptor subsites. Although the acceptor and cyclodextrin binding sites overlapped, the registration of the glucose residues differed. This helps explain the unexpectedly orthogonal orientation of cyclodextrins (Fig. 2) and unproductively bound maltopentaose (Fig. 3) relative to the donor binding site.

We have previously shown that GlgE preferentially extends malto-oligosaccharides with a length of at least four and an optimum of six residues (19). This fits well with the observation that the +3 to +6 acceptor subsites have a particularly high a propensity to bind oligosaccharides including α-cyclodextrin, β-cyclodextrin (19), and unproductively bound maltopentaose (Fig. 3). This is supported by another structure that was obtained with 2-deoxy-2-fluoro-α-maltosyl fluoride and maltohexaose that showed the presence of electron density associated with glucose residues occupying the +2 to +4 subsites along with a maltosyl-enzyme intermediate and five glucose residues in the secondary site (data not shown). This implies that interactions of sugar residues with subsites +1 and +2 are relatively weak but are bolstered with malto-tetraose making additional interactions with subsites +3 (stacks with Trp-448) and +4 (stacks with Tyr-445) to allow productive binding to occur.

The anomeric hydroxyl groups of the sugar residues in the ≥+3 subsites project into solvent. With little or no encroachment of protein, each of these subsites is potentially capable of accommodating an A chain connected to a 6-hydroxyl emanating from a B chain. By contrast, the glucose residues in subsites +1 to +5 have their O6 hydroxyl groups interacting with the surface of the protein (Figs. 4 and 5). This has significant implications for the type of chain GlgE is capable of extending. Polymers consist of linear A, B, and C chains of length ∼7–9 with branches emanating from B and C chains typically halfway along their lengths (Fig. 1) (7). GlgE will be able to extend an A chain without hindrance because such a chain has no branches emanating from any of its 6-OH groups. Because both the B and C chains bear branches from 6-OH groups, neither would be able to bind favorably to GlgE if the branches emanated from sugars in subsites +1 to +5 due to clashes with protein. However, if the external chain length between a branch point and the non-reducing end of a B chain was sufficiently long such that the branch emanated from the ≥+6 subsite, it is feasible that an attached A chain could be accommodated by the linear cleft as indicated in Fig. 4. Similarly, it is possible that the diagonal cleft could accommodate other elements of a polymer that ultimately has a diameter of tens of nm. However, GlgE variants with a disrupted linear cleft did not seem to be kinetically compromised when acting on α-glucan polymer, leaving any potential role for the linear cleft unclear.

Consistent with these observations, we have previously shown that GlgE preferentially extends A chains, resulting in most B chains remaining short and bearing only one branch (7). The acceptor binding site of GlgE contrasts with a much more open one that accommodates only two glucose residues in a classical glycogen synthase (27). This allows B and C chains in classical glycogen to be more readily extended and made available for additional branching by GlgB (28).

The only GlgE variant to have a significantly different *k*_cat_^app^ was the one with the amino acid substitution adjacent to the +1 subsite at His-397 (Table 1). This is consistent with the orientation of the attacking nucleophilic sugar at this subsite needing to be optimal. The unusual conformation of the linkage between the −1 and +1 glucose residues is likely to reflect the appropriate orientation. Interestingly, the +1 pyranose ring is orthogonal to that of the −1 ring such that their respective hydroxyl groups are maximally separated. Perhaps the avoidance of hydrogen bonding interactions between these glucose residues is important in achieving the optimal orientation.

Having already obtained the structure of GlgE with a trapped β-maltosyl intermediate (18), we attempted to obtain such a structure with an acceptor also bound. Despite repeated attempts, we were unable to obtain a ternary Michaelis complex with both a maltosyl-enzyme intermediate and the +1 subsite occupied. It therefore remains to be seen whether such a structure would reveal a ^4^H_3_-type conformation of the −1 sugar ring predicted to promote nucleophilic displacement of the catalytic Asp-394 residue. Distortion could potentially be mediated through the H-bonding interaction between the O6 hydroxyl of the +1 sugar ring with the hydroxyl group of Tyr-357 of the B domain, which forms a lid over the donor binding site. Structural changes like this could provide a mechanism through which GlgE could gate maltosyl transfer to an oligosaccharide rather than to water, analogous to gating in other glucosyltransferases (29).

Finally, product release would presumably require the domain B lid to open. Indeed, the elevated *B* factors of this lid in the product-bound structure are consistent with this likelihood. Tantalizingly, a structure of *M. thermoresistibile* GlgE with maltose bound shows a different conformational state of the S domain together with a loss of electron density of the adjacent B domain of the neighboring subunit, suggesting disorder in the lid and the existence of an open conformation (21).

##### Secondary Binding Site

As discussed above, the linear and diagonal clefts could accommodate elements of the polymer to allow non-reducing ends to gain access to the acceptor site. Their apparent inability to specifically recognize and bind malto-oligosaccharides may simply reflect the need for these spaces to be available for a wide variety of different α-glucan substructures. By contrast, we have identified a new secondary binding site on domain C. This site appears capable of binding either linear or branched oligosaccharides (Figs. 5, 6, and 9). Incidentally, there are only a few other protein structures with branched oligosaccharides bound thus far reported (30, 31).

A likely role for this secondary site is to guide the polymer toward the active site (32). This possibility is supported by the activity of GlgE with a polymeric acceptor being compromised when the secondary site is disrupted. Surprisingly, whether linear or branched, the reducing end of an oligosaccharide projects between the donor sites of the two subunits of GlgE rather than the expected non-reducing end of an acceptor, so how an acceptor polymer is guided to the donor site remains unclear. There has been no indication that the enzyme is processive or subject to allosteric regulation by carbohydrates, so the secondary binding site is less likely to be involved in such phenomena. Another possible function of the secondary site is to co-locate the enzyme and the polymer within the cell. However, this site is not essential in *Streptomyces* because the bacterium was still able to grow and to generate α-glucan with the same fine structure when the site was disrupted.

A somewhat similar but distinct secondary binding site has also been observed on domain C of *M. tuberculosis* GlgE (17). In both cases, five glucose residues wrap around an aromatic amino acid side chain (*S. coelicolor* Tyr-646 *versus M. tuberculosis* Phe-631). Surprisingly, neither of the two secondary sites is spatially conserved, being over 10 Å apart in the superposed structures. This implies evolutionary pressure for this function to exist even if there is no structural conservation. Although the oligosaccharide appears to be bound to the *M. tuberculosis* enzyme in the reverse orientation, the resolution of this structure is only 3.9 Å. Therefore, it seems feasible that it is in fact in the same orientation as that seen in the *S. coelicolor* enzyme, a possibility acknowledged by the authors of the other study (17).

##### Significance for Therapies against Tuberculosis

GlgE is an interesting enzyme not least because it has been genetically validated as a potential target in therapies for tuberculosis (1). GlgE from *S. coelicolor* shares many properties with the enzyme from *M. tuberculosis* and other mycobacteria (1, 17–19, 21). Although a structure of the *M. tuberculosis* enzyme has been reported (17), the *S. coelicolor* enzyme crystallizes more readily to give higher resolution structures. To mimic the *M. tuberculosis* enzyme better, a V279S amino acid substitution has been made in the *S. coelicolor* donor site (20). Having defined the acceptor site in the *S. coelicolor* enzyme, it is now possible to explore whether it is conserved in the *M. tuberculosis* enzyme. Each of the key amino acid residues of subsites +1 to +4 within domain A are conserved (*e.g.* His-397, Trp-448, Arg-449, Tyr-445, and Pro-428; *S. coelicolor* numbering), whereas those of subsites +5 and +6 in domain N of the neighboring subunit are quite different (Gly-84 and Pro-83 are spatially substituted by Glu and Gln, respectively). It is likely that these differences lie behind the lack of inhibition of the *M. tuberculosis* enzyme by cyclodextrins (19). Although the acceptor site is much more open than that of the donor site, it is nevertheless feasible to develop inhibitors that bind to the acceptor subsites, and our new findings allow this to be done in a more rational way.

## Experimental Procedures

### 

#### 

##### Protein Production and Enzyme Assays

Recombinant His-tagged *S. coelicolor* GlgE isoform 1 was produced in *Escherichia coli* and purified using nickel affinity and size exclusion chromatographies as described previously (1, 18, 19). Variants were generated using a QuikChange Lightening site-directed mutagenesis kit (Agilent Technologies) as described previously (18). GlgE activity was determined in triplicate by monitoring the production of inorganic phosphate using malachite green as described previously (1, 18, 19). Maltohexaose concentrations up to 2 mm and α-glucan up to 2.5 mg ml^−1^ were used, ranges in which data conformed to the Michaelis-Menten equation. The α-glucan was isolated from *S. venezuelae* as described previously (7). The values of *k*_cat_ and *K_m_* are apparent because the enzyme conforms to a ping-pong (also known as substituted enzyme) kinetic mechanism (1). Errors reflect S.E. of the fit. Synthetic α-glucan was generated using GlgE and GlgB with α-maltose 1-phosphate and maltohexaose as described previously (7). Linear lengths of constituent chains were determined by treating polymers with isoamylase and analyzing the products using mass spectrometry as described previously (7). The degree of digestion of polymer by β-amylase was determined spectrophotometrically using 3,5-dinitrosalicylic acid where the control sample was completely digested with α-amylase and amyloglucosidase as described previously (7). Isolated α-glucan particles were imaged using a Tecnai 20 transmission electron microscope (FEI, Eindhoven, The Netherlands) at 200 kV with an AMT XR60B digital camera and a uranyl acetate negative stain as described previously (7).

##### Crystallography

Crystals of *S. coelicolor* GlgE isoform 1 were obtained by vapor diffusion from 15% (w/v) polyethylene glycol 3350, 0.2 m sodium citrate, and 15% (v/v) ethylene glycol as described previously (18, 19). For the E423A variant, complexes were obtained by soaking the crystals in crystallization solution containing 5 mm ligand for 5 min prior to mounting. In the case of the malto-octaose product-bound structure, both malto-octaose and 2-deoxy-2-fluoro-α-maltosyl fluoride were present in the soaking solution, but only the former was visible in the resultant complex. For the D394A variant, the ligand-bound structure was obtained by co-crystallization with 5 mm maltodextrin. Crystals were flash cooled in LithoLoops (Molecular Dimensions) by plunging into liquid nitrogen and stored in Unipuck cassettes (MiTeGen) prior to transport to the synchrotron. The crystals were subsequently transferred robotically to the goniostat on station I04-1 or I24 at the Diamond Light Source (Oxfordshire, UK) and maintained at 100 K with a Cryojet cryocooler (Oxford Instruments). Diffraction data were recorded using a either a Pilatus 2 M or a 6 M detector (Dectris) and processed using the xia2 expert system (33). X-ray data collection statistics are summarized in Table 2. The data sets for the E423A variant complexes were isomorphous with that previously collected for the same variant complexed with maltose (Protein Data Bank accession code 4CN6) (18). Therefore, the latter was used as a starting point for modeling these structures, which were completed through several iterations of refinement in REFMAC5 (34) and manual adjustment in Coot (35). In the latter stages, translation libration screw (TLS)^2^ refinement was used with a total of eight TLS domains, which were defined using the TLS motion determination server (36). By contrast, the data set derived from co-crystallization of the D394A variant with maltodextrin was not isomorphous with the above data sets (or any other data set previously obtained for this protein) despite belonging to the same space group and having similar cell parameters. This structure was solved by molecular replacement with Phaser (37) using one subunit taken from the wild-type structure of GlgE isoform 1 co-crystallized with α-cyclodextrin (Protein Data Bank accession code 3ZST) (19) as the starting template. This gave two molecules per asymmetric unit forming the familiar homodimer but having distinctly different crystal contacts presumably dictated by the binding of branched malto-oligosaccharides to the secondary surface binding site. This starting model was refined as for the two complexes described above. Refinement statistics are summarized in Table 2. Simulated annealing omit electron density maps were calculated for the various ligands as follows. The ligands were deleted from the coordinates of the final models, and these were used as inputs to simulated annealing refinement with PHENIX (38) from a starting temperature of 5000 K after applying small random shifts to the models (“shake” term set to 0.3). The resultant *mF*_obs_ − *dF*_calc_ difference electron density maps are shown in Figs. 3, 4*A*, and 6. All structural figures were prepared using CCP4mg (39).

##### Microbiology

The *glgE*-null mutant of *S. venezuelae* ATCC10712 was generated using the Redirect PCR targeting method (40) where the coding region of *glgE* was replaced with an apramycin resistance cassette as described previously (9). The mutant was complemented with *glgE* cloned into the plasmid pMS82 (41) as described previously (9) except that *glgE* was first mutated using the QuikChange kit to allow substitution of Tyr-665 with Ala (*S. venezuelae* numbering). Strains were grown for 2 days on maltose/yeast extract/malt extract/John Innes Centre borehole water 2 solid medium (42) at 28 °C as described previously (9). Colonies were sectioned, stained for α-glucan using periodic acid-thiocarbohydrazide-silver proteinate, and imaged using transmission electron microscopy as described previously (9). α-Glucan was isolated from the complemented strain as described previously and analyzed as described above (7).

## Author Contributions

S. B. coordinated the study and wrote the paper. K. S., C. E. M. S., and D. M. L. edited the manuscript. K. S., M. T., A. G., and A. M. R. carried out the kinetic experiments summarized in Table 1. K. S., C. E. M. S., and D. M. L. carried out the crystallography. J. E. B. carried out the electron microscopy. K. S. and F. M. carried out the microbiology. All authors approved the final version of the manuscript.

## Footnotes

2 The abbreviation used is:

TLS: translation libration screw.
